# Case Report: Severe autoimmune hemolytic anemia in an elderly patient caused by warm-reactive IgG and IgA autoantibodies

**DOI:** 10.3389/fimmu.2025.1664498

**Published:** 2025-11-06

**Authors:** Wenxia Xia, Jialing Lu, Junjie Hou, Jihao Zhou, Haiqing Lin, Xiaoxuan Lai, Xinyou Zhang, Ruiting Zhang, Peng Ke

**Affiliations:** 1 Institute of Transfusion Medicine, Shenzhen Blood Center, Guangdong Shenzhen, China; 2 Shanghai Municipal Hospital of Traditional Chinese Medicine, Shanghai University of Traditional Chinese Medicine, Shanghai, China; 3 Department of Hematology, Shenzhen People’s Hospital, Second Clinical Medical College of Jinan University, First Affiliated Hospital of Southern University of Science and Technology, Shenzhen, China

**Keywords:** autoimmune hemolytic anemia, IgA autoantibody, IgG autoantibody, extended DAT, combination therapy

## Abstract

**Background:**

Autoimmune Hemolytic anemia (AIHA) a relatively uncommon form of hemolytic anemia, which is characterized by the presence of autoantibodies directed against erythrocyte surface antigens, most frequently of the IgG isotype. A positive Direct Antiglobulin Test (DAT) is a key diagnostic criterion for AIHA. However, when hemolysis involves multiple autoantibodies, the standard DAT (polyspecific, anti-IgG + C3) may fail to detect certain antibodies, potentially delaying appropriate treatment.

**Cases presentation:**

We reported one patient with severe AIHA mediated by IgG and IgA autoantibodies was successfully treated with Multi-drug combination regimens. A 58-year-old female was admitted to the hospital presenting with a history of fatigue, jaundice and soy sauce-colored urine for one day. Upon admission, a complete blood count revealed a critically low hemoglobin level of 41 g/L and a life-threatening condition. Initially diagnosed with IgG-mediated AIHA via standard DAT, the patient showed suboptimal response to glucocorticoids, intravenous immunoglobulin (IVIG), and transfusion support. Subsequently, through the extended DAT (monospecific, anti-IgA, anti-IgG, anti-IgM, and anti-C3) test results, the patient was diagnosed as severe AIHA mediated by IgG and IgA. Based on extended DAT results, the treatment plan was modified to include combination therapy with dexamethasone, rituximab, cyclosporine, and bortezomib, alongside intensified plasma exchange.

**Conclusions:**

The extended DAT testing is recommended for all patients with clinical and laboratory evidence of acute hemolysis. Early detection helps in avoiding further investigations and provide efficient management. Severe AIHA mediated by multiple autoantibodies requires early intensive combination therapy, including immunosuppressive agents, IVIG and plasma exchange.

## Introduction

Autoimmune hemolytic anemia (AIHA) is an acquired condition characterized by autoantibodies directed against erythrocyte surface antigens causing destruction of red blood cells (RBCs). The exact incidence of AIHA in adults is unclear, while in children under the age of 18, the incidence rate is estimated at 1 to 3 cases per 100,000 per year (1). AIHA can be classified into primary and secondary according to the etiology. However, AIHA also can be subdivided into warm, mixed or cold-reactive subtypes based on the optimal thermal amplitude used to detect anti-erythrocyte antibodies (2).

Warm AIHA (wAIHA) is the most prevalent form of AIHA, which is caused by increased erythrocyte destruction by immunoglobulin G (IgG) autoantibodies, with or without complement activation, accounting for 60% to 70% of all cases (3). However, IgA can also mediate the occurrence of wAIHA, but wAIHA mediated solely by IgA is very rare (4, 5). Presenting symptoms of AIHA can be heterogenous. Typical clinical symptom in patients with AIHA include fatigue, jaundice, briskness of hemoglobin (Hb) decline, and hemoglobinuria. Laboratory investigations often reveal evidence of hemolysis, including elevated lactate dehydrogenase (LDH), reduced haptoglobin, and increased reticulocyte count. The Direct Antiglobulin Test (DAT), also known as the Coombs test, is a crucial diagnostic tool for identifying AIHA by detecting antibodies or complement proteins attached to the surface of RBCs (6, 7).

Clinically, once hemolysis is confirmed, a DAT is essential to determine immune-mediated etiology. The standard DAT (polyspecific, anti-IgG + C3) demonstrates that IgG and/or complement binding but does not detect IgM or IgA, leading to a negative result in AIHA mediated by these antibodies (8). A particular diagnostic challenge arises when AIHA is mediated by both IgG and IgA autoantibodies simultaneously. Here, the standard DAT is positive due to IgG, potentially masking the concomitant IgA autoantibodies. As AIHA associated with IgA is a rare subtype with a distinct mechanism and often greater treatment difficulty, this oversight can significantly impact outcomes. To overcome the limitations of standard DAT, extended DAT (monospecific, anti-IgG, anti-IgA, anti-IgM, and anti-C3d) is critical for comprehensive serological characterization, and can inform subsequent treatment selection, which ranges from corticosteroids and immunosuppressive agents to novel therapies such as rituximab (9). Here, we present a patient with wAIHA who showed DAT positivity for both IgG and IgA, highlighting the diagnostic and therapeutic challenges encountered in this highly unusual case.

## Case presentation

A 58-year-old female, who presented with fatigue and yellowing of skin for one days, was admitted to our hospital. Complete blood count showed Hb of 41 g/L, MCV 118.9 fl, MCH 36.9 pg, MCHC 311 g/L, absolute reticulocyte count (RET#, 3.8 × 10^9^/L), platelets (PLT, 182 × 10^9^/L) and normal white blood cells (WBC, 4.62 × 10^9^/L) counts. Peripheral blood (PB) smear showed red blood cell agglutination with increased spherocytes, polychromasia, and nucleated red blood cells, but no fragmented red cells. Clinical biochemistry results included total bilirubin = 73.3 μmol/L (normal 1.7-20.0), indirect bilirubin = 63.4 μmol/L (normal 1.7-16.0), lactate dehydrogenase (LDH) = 1107 U/L (normal <247), haptoglobin (HPT) < 0.2 g/L (normal 0.3-2.0), and standard DAT was positive (polyspecific, anti-IgG, and anti-C3). Initial investigations are shown in **Table 1**. The complete blood count and clinical biochemistry of the patient were completely normal 33 days before admission. Furthermore, the patient had not received any treatment prior to this hospitalization. Investigations for causes of secondary AIHA were negative (Supplementary Material). Based on the examination results above, the patient was diagnosed with AIHA.

**Table 1:** Clinical characteristics and laboratory results of the patient.

Inspection date	Initial diagnosis (Day 0)	Day 6	Day 30	Day 41	Day 106
Laboratory test	Result	Result	Result	Result	Result
Hemoglobin and red blood cell indices	Hb 41g/L, MCV 118.9 fl, MCH 36.9pg	Hb 60g/L, MCV 91 fl, MCH 32.6pg	Hb 97g/L, MCV 118 fl, MCH 39.4pg	Hb 98g/L, MCV 109.8 fl, MCH 46.4pg	Hb 109g/L, MCV 104.8 fl, MCH 36.1pg
Anemia detection	Ferritin 467.0 µg/L (normal 11.0-306.8), Folic acid 29.1 nmol/L (normal 7.0-45.1), Vitamin B12–186 pmol/L (normal 133-675)				
Hemolysis panel	T-Bil = 73.3 µmol/L (normal 1.7-20.0), I-Bil = 63.4 µmol/L (normal 1.7-16.0), LDH = 1107 U/L (normal <247), HPT < 0.2 g/L (normal 0.3-2.0)	T-Bil = 77.1 µmol/L, I-Bil = 67.6 µmol/L, LDH = 637 U/L, HPT < 0.2 g/L	T-Bil = 30.3 µmol/L, I-Bil = 25.1 µmol/L, LDH = 354 U/L, HPT < 0.2 g/L	T-Bil = 35.3 µmol/L, I-Bil = 29.2 µmol/L, LDH = 387 U/L, HPT < 0.2 g/L	T-Bil = 21.3 µmol/L, I-Bil = 11.2 µmol/L, LDH = 251 U/L, HPT = 0.4 g/L
Inflammatory markers	CRP 4.1 mg/L (normal 0-6.0), PCT 0.06 ng/mL (normal <0.05), IL-6 4.9 pg/mL				
Direct antiglobulin test	IgG autoantibody + C3 (4+ strength), IgA and IgM autoantibodies were not detected	IgG autoantibody (4+ strength), IgA autoantibody (4+ strength),C3 complement and IgM were negative	IgG autoantibody (3+ strength), IgA autoantibody (2~3+ strength),C3 complement and IgM were negative	IgG autoantibody (2+ strength), IgA autoantibody (2+ strength),C3 complement and IgM were negative	Not detected

Hb, hemoglobin; MCV, mean corpuscular volume; MCH, mean corpuscular hemoglobin; T-Bil, Total bilirubin; I-Bil, Indirect bilirubin; LDH, lactate dehydrogenase; CRP, C-reactive protein; PCT, procalcitonin; C3, Complement Component 3.

**Figure 1:**
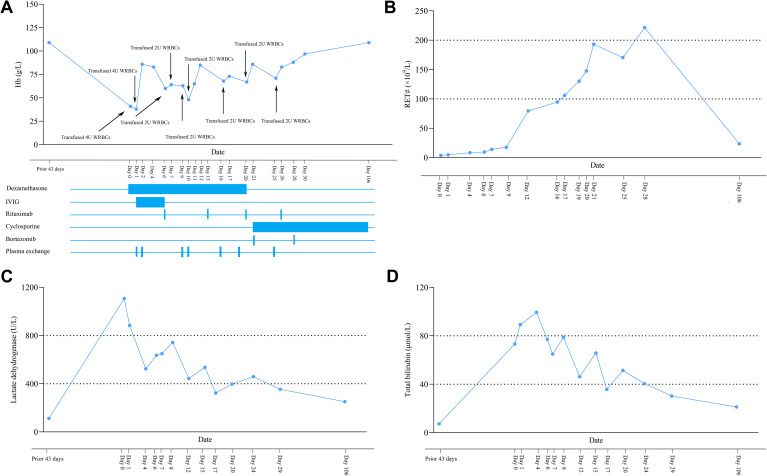
Laboratory value trends (**(A)**, hemoglobin concentration; **(B)**, absolute reticulocyte count; **(C)**, lactate dehydrogenase; and **(D)**, total bilirubin) throughout hospital course. Time points (arrows) of clinical intervention are indicated in **(A)**.

The patient started dexamethasone (0.75mg/kg daily) treatment on the day of admission, and received 4 units washed red blood cells infusion on the same day. However, the patient’s routine blood results one day after admission showed the Hb of only 38 g/L and a further elevation of total bilirubin (89.2 μmol/L). On that day, a plasma exchange and 4 units washed red blood cells infusion were administered. Meanwhile, a single dose of human immunoglobulin (IVIG) at 0.4g/kg was given continuously for 5 days. Two days after admission, the patient’s Hb rose to 83 g/L and remained around 80 g/L for the next few days. However, six days after admission, Hb dropped again to 60 g/L. The extended DAT panel was used to retest the patient’s autoantibodies. IgA autoantibody (4+ strength) and IgG autoantibody (4+ strength) were identified on the erythrocytes, but no surface complement. On the same day, considering the detection of IgA autoantibodies and the suboptimal response to conventional treatment, we decided to administer rituximab at a fixed dose of 375 mg/m² weekly for 4 weeks. During the initial administration of rituximab, the patient experienced a grade 2 infusion reaction. After reducing the infusion rate and administering anti-allergic and anti-inflammatory medication, the symptoms resolved. No subsequent infusion reactions occurred during following rituximab administrations. Meanwhile, washed red blood cells transfusions and plasma exchange were performed intermittently. Twenty days after admission, the patient’s Hb was only 65 g/L, and bilirubin and LDH levels increased compared with before. Considering the recurrence of the patient’s condition, cyclosporine (100 mg twice daily orally) and bortezomib (1.3 mg/m² weekly subcutaneously) were added to the treatment regimen, and dexamethasone was discontinued concurrently. Meanwhile, intermittent infusion of washed red blood cells and plasma exchange were continued. From twenty-four to thirty days after admission, without blood transfusion, the patient’s Hb gradually increased from 71 g/L to 97 g/L, IgA (2~3+ strength) and IgG (3+ strength) autoantibodies were significantly reduced relative to baseline. Thirty days after admission, the patient requested discharge and was prescribed cyclosporine (100 mg orally twice daily) for outpatient continuation. The first follow-up was conducted 41 days after admission, at which time the patient continued oral cyclosporine treatment. The Hb level remained stable at 98 g/L; however, IgA and IgG autoantibodies (both 2+ intensity) remained detectable via gel card testing. Subsequent follow-ups were conducted weekly. At the final follow-up 106 days after admission, the patient was still receiving oral cyclosporine (100 mg orally twice daily). The Hb level had increased to 109 g/L, and both IgA and IgG antibodies were no longer detectable. The patient’s clinical course, including interventions and significant laboratory value trends, is depicted in **Figure 1**. Additionally, detailed documentation regarding this patient’s blood transfusion management has been provided in the Supplementary Material.

## Discussion

The standard DAT is routinely done using poly-specific anti human globulin antisera, which consists of anti-IgG and anti-C3d antibodies (10). Positive DAT is one of a crucial diagnostic criteria AIHA, but there is also DAT-negative immune hemolytic anemia. Previous studies have established that DAT-negative AIHA accounts for 7.8% of all AIHA cases and 11% of wAIHA cases (11). However, the standard DAT method fails to accurately detect autoantibody presence. When hemolysis is mediated by IgA or IgM autoantibodies, this test typically yields negative results. When hemolytic anemia is mediated by multiple autoantibodies simultaneously, the accuracy of standard DAT detection is further compromised.

In our case, the patient’s hemolysis was mediated by both IgG and IgA autoantibodies; However, the initial standard DAT positivity led us to ignore the presence of IgA autoantibodies. Unlike the standard DAT method, the extended DAT method utilizes five monospecific anti-human globulin reagents (anti-IgG, anti-IgA, anti-IgM, anti-C3c, and anti-C3d) suspended in gel with an appropriate negative control, significantly improving autoantibody detection rates. A large-scale study of 9382 AIHA patients demonstrated that the autoantibody detection rates with the extended DAT method was significantly higher than the standard DAT method (100% vs. 50.7%, P<0.01) (12). Therefore, it is highly necessary to conduct extended DAT testing for patients with suspected acute AIHA.

wAIHA is mainly IgG-mediated extravascular hemolysis, which can occur at any age, but there is a slight female predominance in most series. The classical mechanism of IgG autoantibody-mediated immune hemolytic anemia proposes that IgG subtypes activate complement, resulting in C3 fragment deposition on red blood cells. These opsonized red blood cells are subsequently cleared by liver macrophages that carry receptors for C3 fragments (13). However, IgG autoantibodies can also directly bind to red blood cells to produce IgG-coated red blood cells. IgG-coated red cells can be recognized by splenic macrophages through the Fcγ receptor of the IgG heavy chain and eventually be phagocytosed and destroyed (14). Furthermore, extracellular hemolysis in the spleen may also occur via antibody-dependent cell-mediated cytotoxicity from T cells that also expressing Fcγ receptors (13). Similar to IgG autoantibody-mediated hemolysis, IgA autoantibody-mediated hemolysis may involve complement-dependent cytotoxicity or directly bind to red blood cells for subsequent recognition and phagocytosis by macrophages, thereby causing hemolysis (15, 16). In recent years, Chadebech et al. discovered that IgA autoantibodies can directly induce aggregation of mature red blood cells, leading to splenic sequestration and destruction without complement activation or FcαRI involvement (17). In this case, extended DAT test was C3-negative, with normal peripheral blood C3 levels, indicating that hemolysis was independent of complement C3 activation. However, the precise hemolytic mechanism in this patient remains unclear.

Steroids remain the first-line therapy for wAIHA, particularly IgG-mediated wAIHA, and similarly some cases of IgA-mediated wAIHA also respond well to corticosteroid therapy (18). However, some patients with IgA autoantibody-mediated wAIHA require sustained high-dose corticosteroids to maintain remission, and some even exhibit treatment refractoriness and require alternative treatments (19). Based on two randomized controlled trials, rituximab combined with glucocorticoids is also recommended as first-line therapy for wAIHA conditions. One open-label Phase 3 trial randomized 64 patients to receive prednisolone with or without intravenous rituximab (375 mg/m² weekly for 4 weeks). The prednisolone-rituximab group demonstrated a significantly higher response rate than the prednisolone group at 36-month follow-up (70% vs. 45%, P<0.05) (20). In another double-blind trial randomized 32 patients with AIHA who had received prednisone for less than 6 weeks to prednisone-placebo vs prednisone-rituximab (1000 mg on days 1 and 15). Again, the overall response at 12 months of prednisone-rituximab group was significantly higher than prednisone-placebo group (75% vs. 31%, P = 0.032) (21). In addition, rituximab, a well-known chimeric monoclonal antibody targeting the CD20 antigen on B lymphocytes, has demonstrated high efficacy in multiple relapsed/refractory wAIHA studies and is also regarded as one of the most important and effective second-line treatments (22–24). Immunosuppressive agents with favorable safety profiles, such as mycophenolate mofetil, azathioprine, or cyclosporine, are also one of the important options for patients with relapsed/refractory AIHA (25–27). In recent years, due to its ability to induce plasma cell apoptosis, the proteasome inhibitor bortezomib has been employed alone or combined with dexamethasone, rituximab, or plasma exchange for the treatment of AIHA (28–32). Plasma exchange has limited efficacy, but it may provide temporary clinical benefit in fulminant hemolysis until pharmacotherapy becomes effective. Some patients with hemolysis may also benefit from IVIG (33, 34).

In this case, initial treatment with corticosteroids was guided by standard DAT results but yielded a suboptimal response. Following the detection of IgA autoantibodies via extended DAT method, rituximab was added and plasma exchange frequency increased. Following combination therapy including dexamethasone, rituximab, IVIG, and plasma exchange, the patient’s transfusion requirements progressively declined, accompanied by reductions in hemolytic markers and autoantibody titers. Subsequently, to minimize long-term high-dose corticosteroid toxicity, glucocorticoids were discontinued in favor of lower-toxicity agents (cyclosporine and bortezomib). Ultimately, the patient with refractory AIHA achieved remission following multi-drug regimen therapy. Throughout the patient’s multi-drug combination treatment, no treatment-related adverse events, such as infection or gastrointestinal bleeding, were observed. This indicates that when there are protective measures including IVIG and proton pump inhibitors, this protocol is relatively safe.

## Conclusions

We report a rare case of severe AIHA mediated by IgG and IgA autoantibodies that was treated successfully with multi-drug combinations. This case exhibits the main clinical features of AIHA due to warm IgA: idiopathic, rapid progression and severe hemolysis (35). Our case illustrates that standard DAT positivity does not exclusively indicate IgG-mediated AIHA; other autoantibodies, such as IgA, can coexist and contribute to hemolysis. Therefore, extended DAT testing is recommended for all patients with suspected acute autoimmune hemolytic anemia. Additionally, patients with severe AIHA mediated by multiple autoantibodies may require combination therapy with high-dose corticosteroids, rituximab, IVIG, plasma exchange, and even immunosuppressive agents.

## Data Availability

The raw data supporting the conclusions of this article will be made available by the authors, without undue reservation.
