# Supplementary material for: Case Report: Severe autoimmune hemolytic anemia in an elderly patient caused by warm-reactive IgG and IgA autoantibodies
Source: Front Immunol. 2025 Nov 6;16:1664498. doi: 10.3389/fimmu.2025.1664498 (PMC12631332; doi:10.3389/fimmu.2025.1664498)
Supplement: Supplementary file 1 [file Table1.docx]

| **Table S1. Additional Workup of Hemolytic Anemia** | |
| --- | --- |
| **Investigation** | **Result** |
| CT Thorax/Abdomen/Pelvis | No evidence of lymphoma |
| Viral Serologies | HBsAg non-reactive, HBcAb non-reactive, HCV Ab non-reactive, HIV Ab non-reactive, EBV-DNA and CMV-DNA were negative |
| Auto-immune panel | Rheumatic disease-related antibodies (ANA, ENA, and anticardiolipin antibodies) were negative |
| Bone Marrow cell morphology | Decreased bone marrow hyperplasia, increased granulocytic ratio, decreased erythroid and megakaryocytic hyperplasia, and agglutination distribution of mature red blood cells |
| Bone Marrow Flow Cytometry | No cells exhibiting abnormal phenotypes were detected in this assay |
| Bone Marrow Biopsy | The degree of myelodysplasia was low (approximately 30%). Both granulocytic and erythroid cells were predominantly at intermediate maturation stages, and megakaryocytes exhibited predominantly lobulated nuclei. No fibrosis was evident, and there was no evidence of malignancy. |
| Others | The cold agglutination test was negative; The activity of glucose-6-phosphate dehydrogenase was normal; No PNH clones detected |

Abbreviations: CT, computerized tomography; HCV, hepatitis C virus; HIV, human immunodeficiency virus; EBV, Epstein-Barr virus; DNA; deoxyribonucleic acid; CMV, cytomegalovirus; ANA, antinuclear antibody; ENA, extracted nuclear antigens.

**Recommended Blood Transfusion Protocol for Patients with AIHA**

**1. General Principle**

Blood transfusion should be avoided or minimized in patients with AIHA whenever possible. The presence of autoantibodies complicates cross-matching procedures and increases the risk of hemolytic transfusion reactions mediated by alloantibodies.

**2. Transfusion Indication**

The decision to transfuse should be based on the severity of anemia, the presence and severity of clinical symptoms, and the rate of hemoglobin decline. For patients with acute hemolytic anemia presenting severe symptoms (e.g., hemodynamic instability, signs of end-organ ischemia), transfusion should be administered without delay.

**3. Blood Product Selection**

- No alloantibodies were detected in our patient's serum, so red blood cells with the same ABO, Rh and Kidd phenotypes and less antigen specificity were preferred;
- Cross-matching tests were performed alongside self-controls；
- Select donor red blood cells that exhibit agglutination no stronger than that observed in the self-control or the weakest reaction among the tested units.

**4. Transfusion Procedure**

Administer blood products slowly with close monitoring for any signs of transfusion reactions. Prophylactic glucocorticoids may be administered before transfusion to reduce the incidence and severity of transfusion reactions.
